# Supplementary material for: Adenosine-generating CD39+ plasmablasts predispose to successful infliximab therapy in pediatric IBD
Source: Life Sci Alliance. 2025 Apr 8;8(6):e202403055. doi: 10.26508/lsa.202403055 (PMC11979362; doi:10.26508/lsa.202403055)
Supplement: Supplementary file 1 [file LSA-2024-03055_TableS1.docx]

# Supplementary Table 1

**Supplementary Table 1 – List of used antibodies**

| **Antigen** | **IgG-Subclass** | **Clone** | **Conjugate** | **Producer** | **Catalogue #** |
| --- | --- | --- | --- | --- | --- |
| CD24 | mourse, IgG1 k | SN3 A5-2H10 | FITC | eBioscience | 11-0247-42 |
| CD24 | mourse, IgG2a k | ML5 | BB515 | BD | 564521 |
| beta-7 | rec. human IgG1 | REA441 | PE | Miltenyi | 130-106-440 |
| CCR9 | mourse, IgG1 k | BBC3M4 | PE | eBioscience | 12-1999-42 |
| CD62-L | mourse, IgG1 k | DREG-56 | PE | BD | 555544 |
| CXCR3 | mourse, IgG1 | 1C6 | PE | BD | 557185 |
| IL10 | rat, IgG1 | JES3-9D7 | PE | eBioscience | 12-7108-82 |
| CD20 | mouse, IgG2b κ | 2H7 | PerCP-Cy5.5 | eBioscience | 45-0209-42 |
| TNF | mourse, IgG1 k | MAb11 | PerCP-Cy5.5 | BD | 560679 |
| CD38 | mourse, IgG1 k | HB-7 | PE-Cy7 | BD | 356608 |
| CCR5 | rec. IgG1 | REA245 | APC | Miltenyi | 130-106-224 |
| CD49d | rec. IgG1 | REA545 | APC | Miltenyi | 130-108-231 |
| beta-7 | rec. IgG1 | REA441 | APC | Miltenyi | 130-106-441 |
| CCR6 | rec. IgG1 | REA190 | APC | Miltenyi | 130-100-373 |
| CXCR5 | mourse, IgG2b k | RF8B2 | AF 647 | BD | 558113 |
| Granzyme B | rec. IgG1 | REA226 | APC | Miltenyi | 130-101-346 |
| CD138 | mourse, IgG1 k | MI15 | BV421 | BD | 356516 |
| CD27 | mourse, IgG1 k | L128 | BV510 | BD | 563092 |
| FVS780 |  |  | APC-Cy7 | BD | 565388 |
